# Supplementary material for: Human-induced intensified seasonal cycle of sea surface temperature
Source: Nat Commun. 2024 May 10;15:3948. doi: 10.1038/s41467-024-48381-3 (PMC11087490; doi:10.1038/s41467-024-48381-3)
Supplement: Supplementary file 1 — Supplementary Information [file 41467_2024_48381_MOESM1_ESM.pdf]

**Supplementary Information**

# Human-induced intensified seasonal cycle of sea surface temperature

Fukai Liu[1*], Fengfei Song[1,2*], Yiyong Luo[1*]

1. Frontiers Science Center for Deep Ocean Multispheres and Earth System and Physical Oceanography Laboratory, Ocean University of China, Qingdao, China
2. Laoshan Laboratory, Qingdao, China

Correspondence to: Fukai Liu (fliu@ouc.edu.cn), Yiyong Luo (yiyongluo@ouc.edu.cn), and Fengfei Song (songfengfei@ouc.edu.cn)

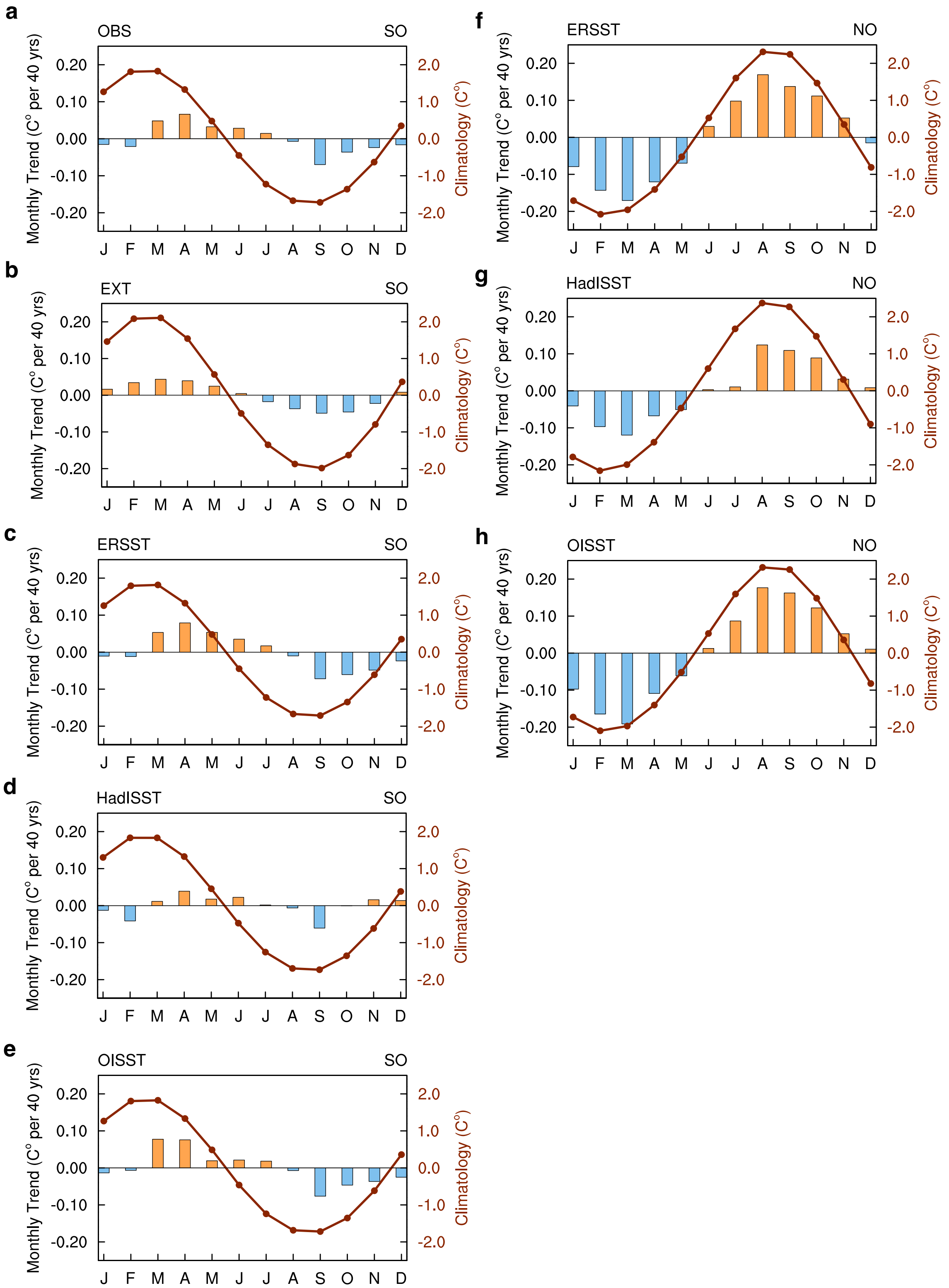


**Supplementary Fig. 1 | Observed and simulated changes in SST seasonality. a-e,** Same as Fig. 1**a,** but for SST averaged over the Southern Hemisphere (0-60 °S) in (a) OBS, (b) EXT, (c) ERSST, (d) HadISST, and (e) OISST. **f-h,** Same as Fig. 1**a**, but for the (f) ERSST, (g) HadISST, and (h) OISST datasets.

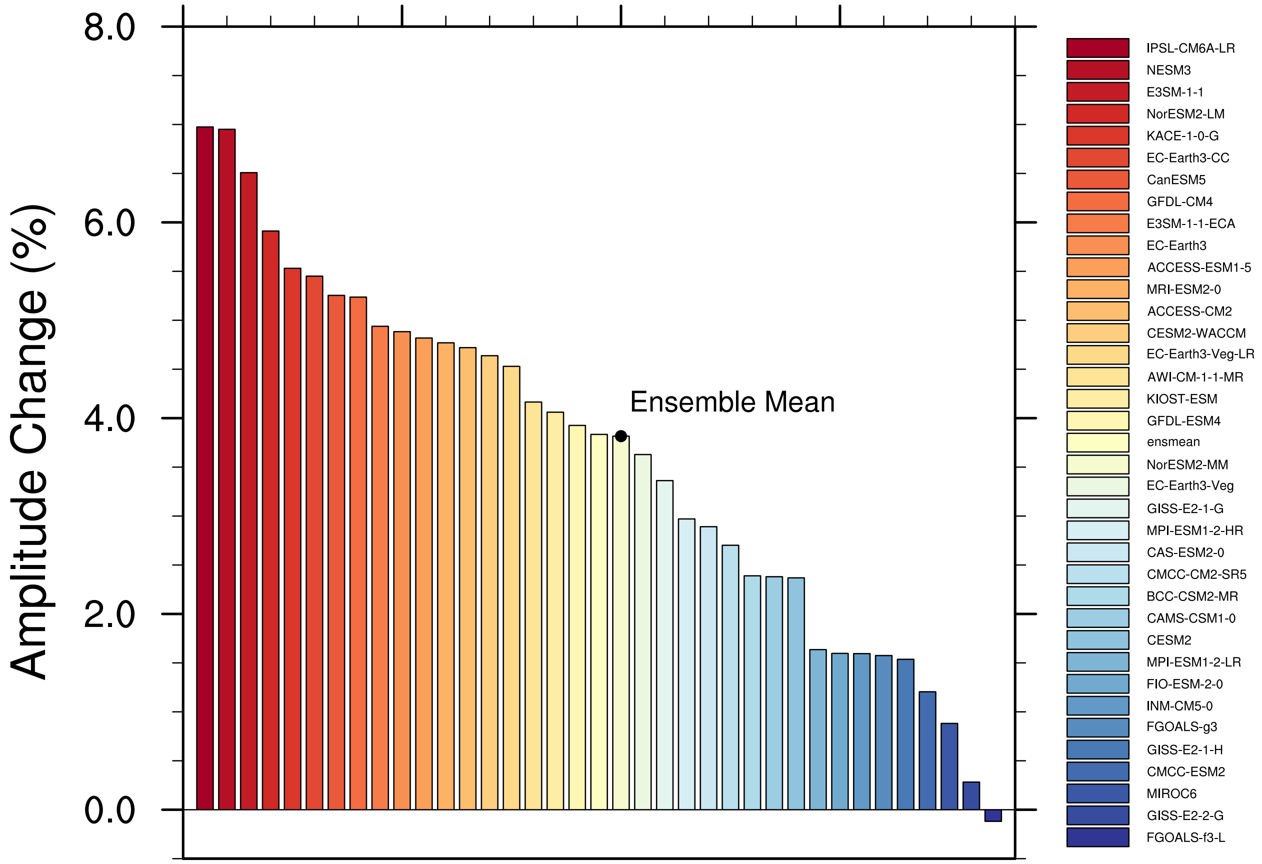


**Supplementary Fig. 2 | Consistency of intensified SST seasonal cycle among different CMIP6 models.** Trends of the global mean amplitude of the SST seasonal cycle (unit: % per 40yr) from 1983 to 2022 in all 36 CMIP6 models.

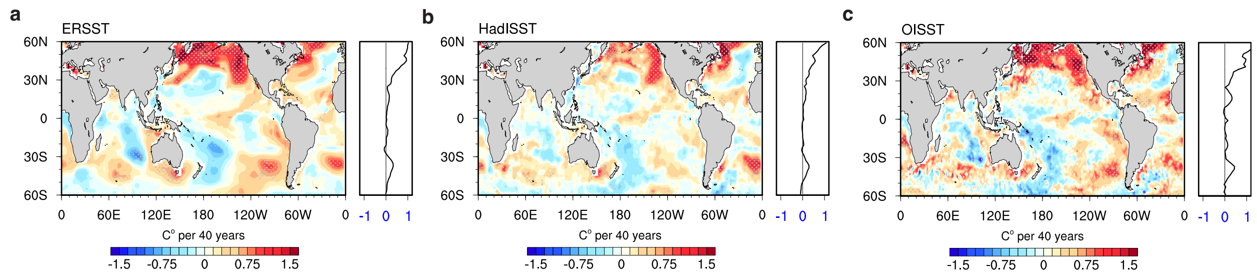


**Supplementary Fig. 3 | Observed patterns of amplitude changes in the SST seasonal cycle. a-c,** Same as Fig. 1**e**, but for (a) ERSST, (b) HadISST, and (c) OISST datasets.

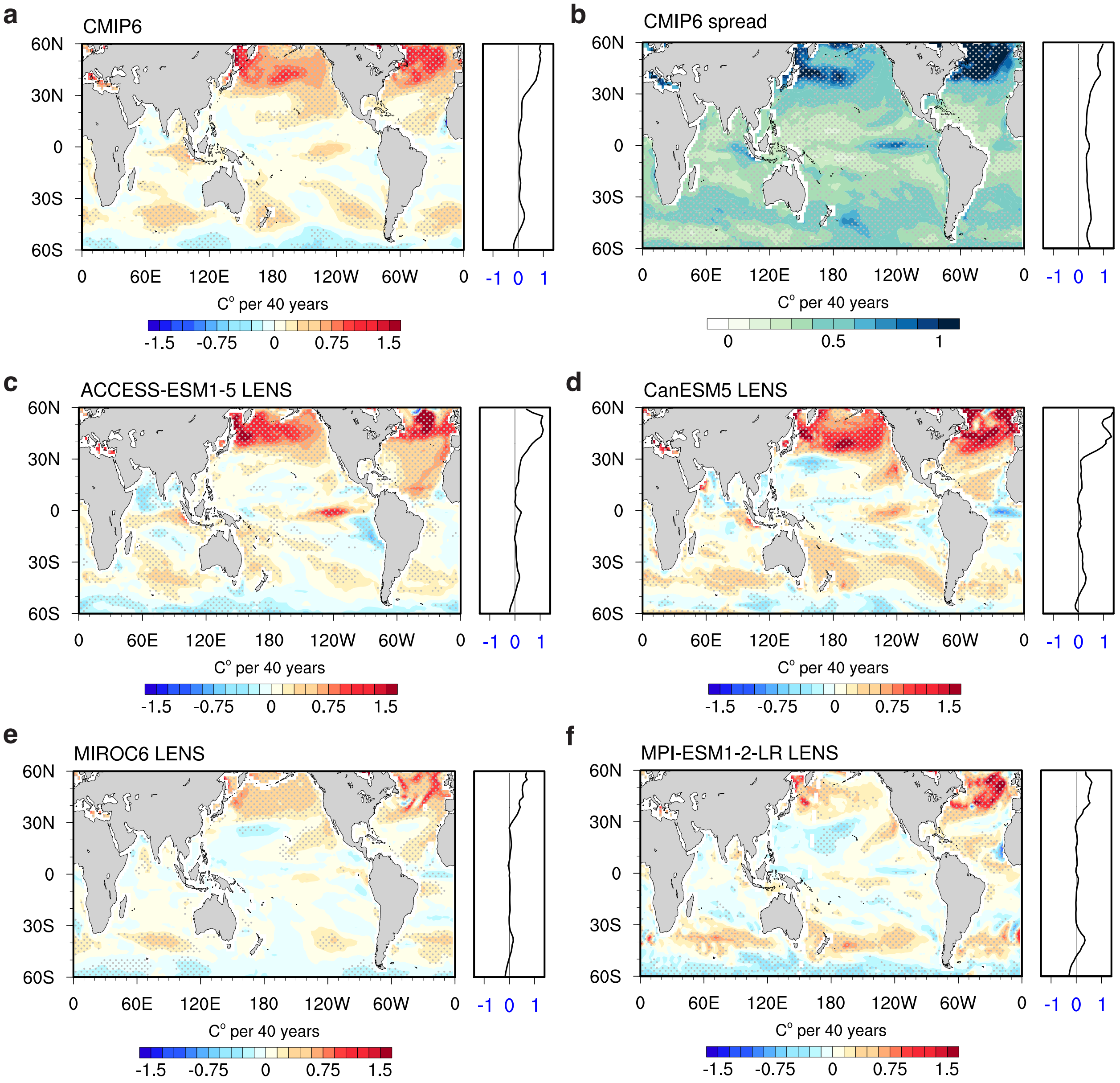


**Supplementary Fig. 4 | Simulated patterns of amplitude changes in the SST seasonal cycle.** **a**, Same as Fig. 1**f**, but for (**a**) the CMIP6 multi-member ensemble (MME) mean. Stippling indicates where the trend is statistically significant above the 95% confidence level based on Student's *t* test. **b**, Ensemble standard deviation of linear trends of SST seasonal cycle amplitude among CMIP6 MME. Stippling indicates where the difference between OBS and CMIP6 MME mean is smaller than the ensemble standard deviation. **c-f,** Same as **a**, bur for (**c**) the mean of ACCESS-ESM1-5 LENS, (**d**) the MME of CanESM5 LENS, (**e**) the MME of MIROC6 LENS, and (**f**) the MME of MPI-ESM1-2-LR LENS.

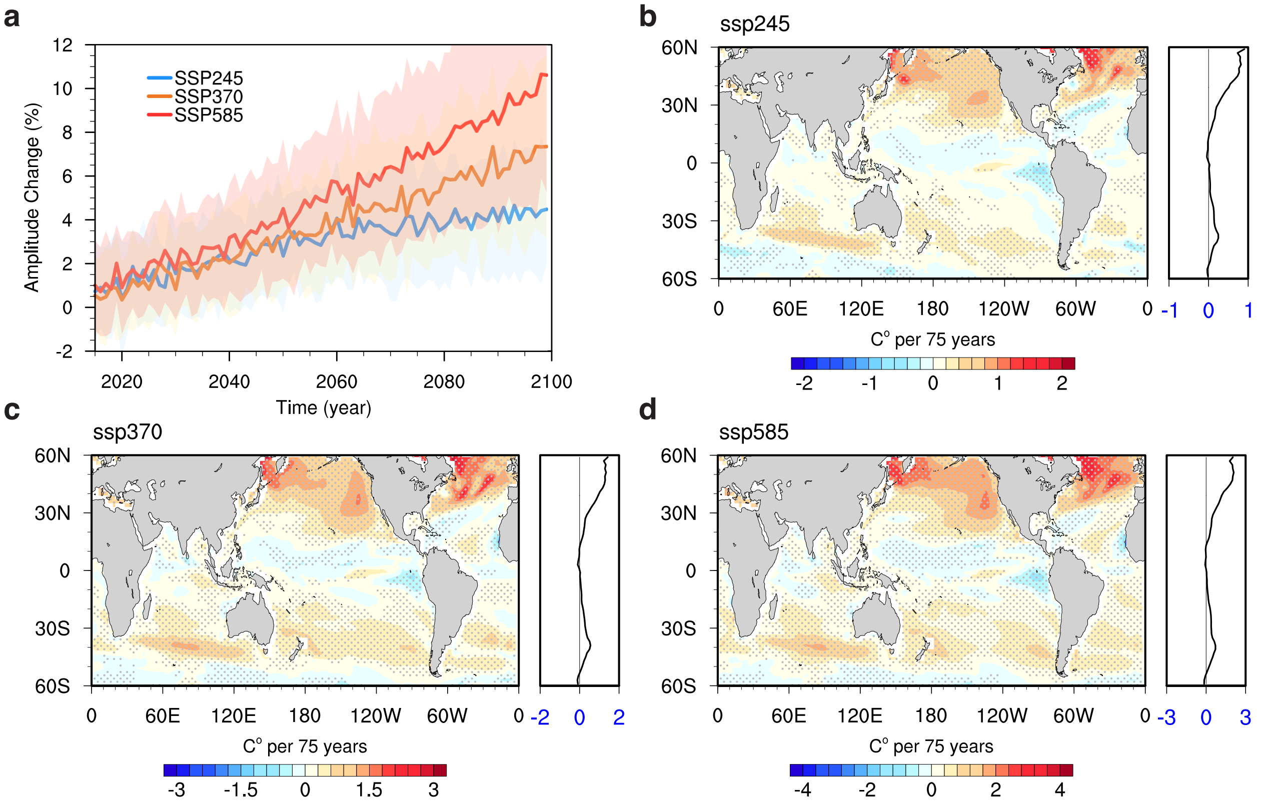


**Supplementary Fig. 5 | Projected increased amplitude of SST seasonal cycle in the future climate. a**, The temporal evolution of the global mean amplitude of the SST seasonal cycle (unit: %) in the SSP2-4.5 (red), SSP3-7.0 (orange), and SSP5-8.5 (blue) scenarios of the CMIP6 simulations. Shadings indicate one standard deviation from the multi-model means. **b**, Linear trend of SST seasonal cycle amplitude (unit: °C per 75yr) during 2025-2099 from the SSP2-4.5 scenario, with its zonal mean shown in the right-hand panel. **c, d**, Same as **b**, but for the SSP3-7.0 and SSP5-8.5 scenarios. Stippling in **b-d** indicates where the trend is statistically significant above the 95% confidence level based on Student's *t* test.

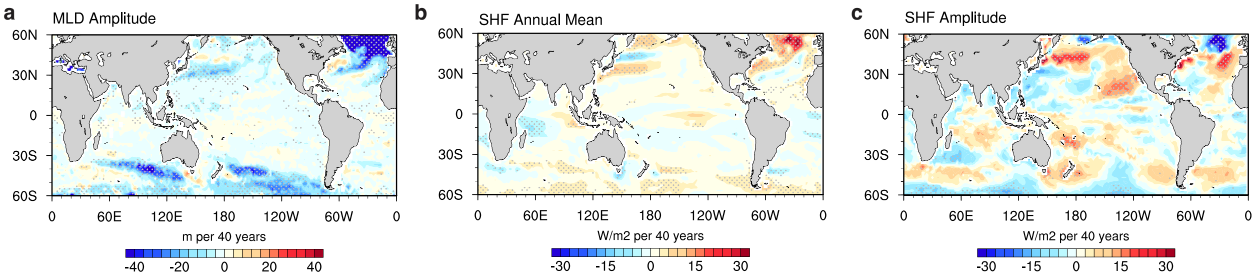


**Supplementary Fig. 6 |** Linear trend of (a) seasonal amplitude of MLD, (b) annual mean SHF (unit: $W\ m^{-2}$ per 40yr), and (c) seasonal amplitude of SHF (unit: $W\ m^{-2}$ per 40yr) during 1983-2022 from CMIP6-MME. Stippling indicates where the trend is statistically significant above the 95% confidence level based on Student's *t* test.

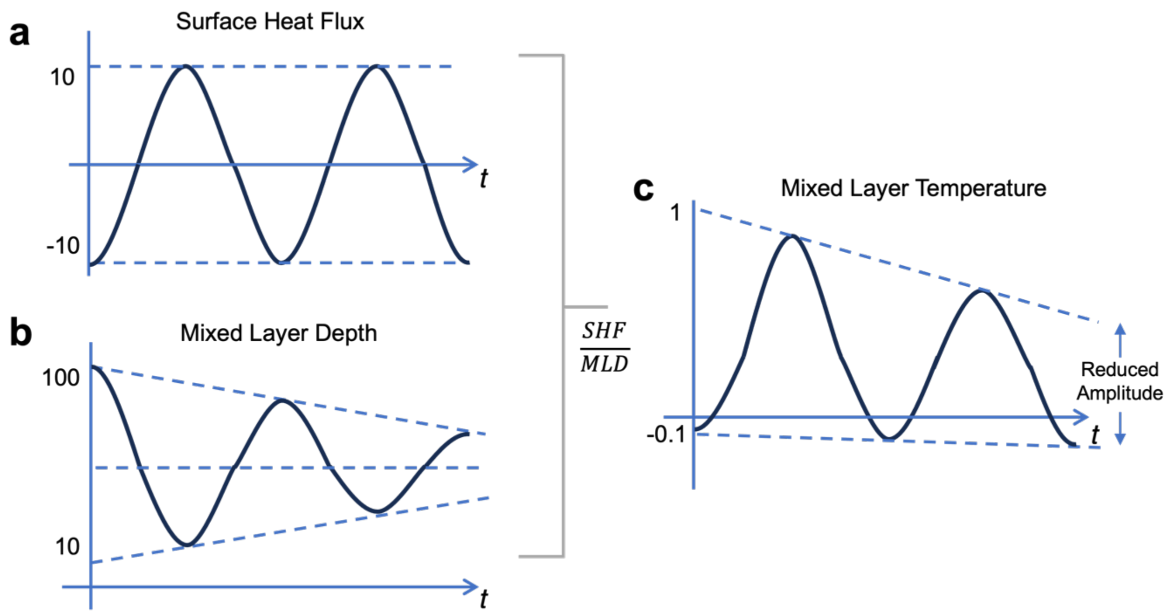


**Supplementary Fig. 7 | Schematic showing how the reduced seasonal amplitude in MLD suppresses the SST seasonality. a,** Annual-repeating SHF without any changes in the seasonal cycle. **b**, MLD with reduced seasonal amplitude but no long-term trend. **c,** Resulting thermal forcing term characterized by decreased seasonal amplitude.

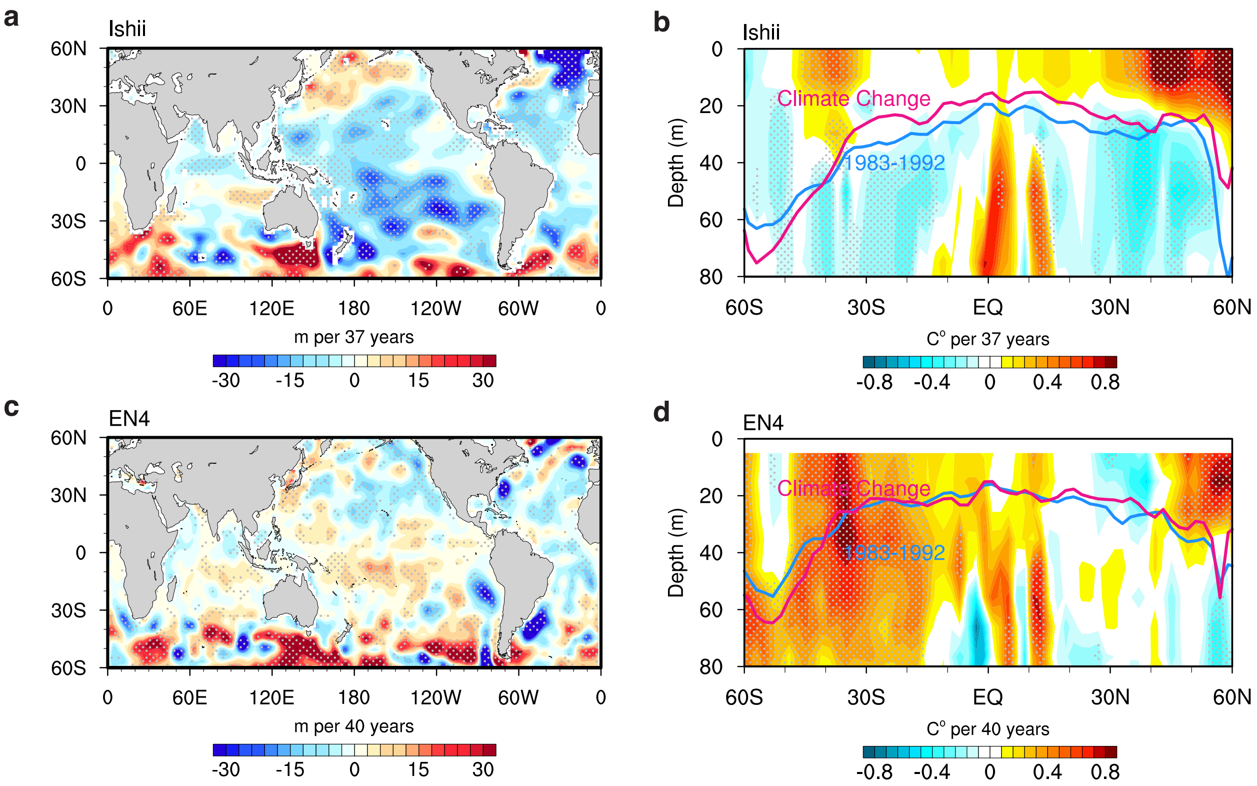


**Supplementary Fig. 8 | Observed trends in annual-mean MLD and amplitude of upper-ocean temperature seasonal cycle. a-d,** Same as Fig. 5**a, b,** but for the (**a, b**) Ishii, and (**c, d**) EN4 datasets. Note that, for the Ishii dataset, the trend is calculated for 1983-2019, and thus the corresponding unit is °C per 37yr.

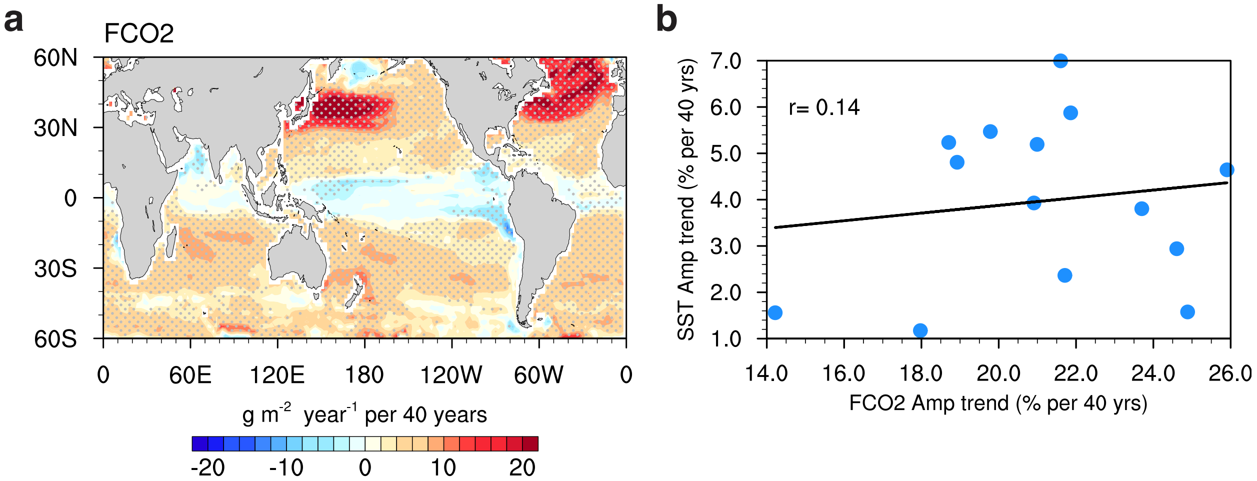


**Supplementary Fig. 9 | Intensified seasonal cycle of surface CO2 flux unrelated to changes in SST seasonal cycle. a,** Linear trend of surface CO2 flux seasonal cycle amplitude (unit: g $m^{-2}$ $year^{-1}$ per 40yr) during 1983-2022 from CMIP6-MME. **b,** an inter-model relationship between the trend of surface dissolved oxygen seasonal cycle amplitude and the trend of SST seasonal cycle amplitude during 1983-2022.

**Supplementary Table. 1 | The CMIP6 model variables used in this study.**

| CMIP6 Model | Sea surface temperature | Mixed layer depth | Surface heat fluxes | Ocean temperature | Surface dissolved oxygen | Air-sea CO2 flux | Ocean current velocity |
|---|---|---|---|---|---|---|---|
| ACCESS-CM2 | H, S2, S3, S5, HG(3), HA(3), HN (3) | H, S5 | H, S5 | | | H, S5 | |
| ACCESS-ESM1-5 | H, S2, S3, S5, HG(3), HA(3), HN (3) | H, S5 | H, S5 | H, S5 | H, S5 | | |
| AWI-CM-1-1-MR | H, S2, S3, S5 | H, S5 | H, S5 | | | | |
| BCC-CSM2-MR | H, S2, S3, S5, HG(3), HA(3), HN (3) | H, S5 | H, S5 | H, S5 | | | |
| CAMS-CSM1-0 | H, S2, S3, S5 | H, S5 | H, S5 | H, S5 | | | H, S5 |
| CAS-ESM2-0 | H, S2, S3, S5 | H, S5 | H, S5 | H, S5 | | | H, S5 |
| CanESM5 | H, S2, S3, S5, HG(10), HA(10), HN (10) | H, S5 | H, S5 | H, S5 | H, S5 | H, S5 | H, S5 |
| CESM2 | H, S2, S3, S5 | H, S5 | H, S5 | H, S5 | | H, S5 | |
| CESM2-WACCM | H, S2, S3, S5 | H, S5 | H, S5 | H, S5 | | H, S5 | H, S5 |
| CMCC-CM2-SR5 | H, S2, S3, S5 | H, S5 | H, S5 | | | | |
| CMCC-ESM2 | H, S2, S3, S5 | H, S5 | H, S5 | | H, S5 | H, S5 | |
| E3SM-1-1 | H, S5 | H, S5 | H, S5 | H, S5 | | | |
| E3SM-1-1-ECA | H, S5 | H, S5 | H, S5 | | | | |
| EC-Earth3 | H, S2, S3, S5 | | | | | | |
| EC-Earth3-CC | H, S2, S5 | H, S5 | H, S5 | | H, S5 | H, S5 | |
| EC-Earth3-Veg | H, S2, S3, S5 | H, S5 | | | | | |

| | | | | | | | |
|---|---|---|---|---|---|---|---|
| EC-Earth3-Veg-LR | H, S2, S3, S5 | H, S5 | | | | | |
| FGOALS-f3-L | H, S2, S3, S5 | H, S5 | H, S5 | H, S5 | | | |
| FGOALS-g3 | H, S2, S3, S5, HG(3), HA(3), HN (3) | H, S5 | H, S5 | H, S5 | | | H, S5 |
| FIO-ESM-2-0 | H, S2, S5 | | H, S5 | H, S5 | | | |
| GFDL-CM4 | H, S2, S5 | H, S5 | | H, S5 | | H, S5 | |
| GFDL-ESM4 | H, S2, S3, S5 | H, S5 | | | H, S5 | H, S5 | |
| GISS-E2-1-G | H, S2, S3, S5 | H, S5 | H, S5 | H, S5 | | | |
| GISS-E2-2-G | H, S2, S3, S5 | H, S5 | | | | | |
| GISS-E2-1-H | H, S2, S3, S5 | | | | | | |
| INM-CM5-0 | H, S2, S3, S5 | | H, S5 | | | H, S5 | |
| IPSL-CM6A-LR | H, S5, HG(8), HA(8), HN (8) | | | H, S5 | H, S5 | H, S5 | H, S5 |
| KACE-1-0-G | H, S2, S3, S5 | | H, S5 | | | | |
| KIOST-ESM | H, S2, S5 | H, S5 | | | H, S5 | | |
| MIROC6 | H, S2, S3, S5, HG(9), HA(9), HN (9) | H, S5 | H, S5 | H, S5 | | | |
| MPI-ESM1-2-HR | H, S2, S3, S5 | H, S5 | H, S5 | | H, S5 | H, S5 | |
| MPI-ESM1-2-LR | H, S2, S3, S5 | H, S5 | H, S5 | | H, S5 | H, S5 | |
| MRI-ESM2-0 | H, S2, S3, S5, HG(5), HA(5), HN (5) | H, S5 | H, S5 | H, S5 | | | |
| NESM3 | H, S2, S5 | H, S5 | H, S5 | H, S5 | | | H, S5 |
| NorESM2-LM | H, S2, S3, S5 | H, S5 | | H, S5 | H, S5 | H, S5 | |
| NorESM2-MM | H, S2, S3, S5 | H, S5 | | | H, S5 | H, S5 | |

"H" denotes the historical simulation; "S2", "S3", and "S5" denote the SSP2-4.5, SSP3-7.0, SSP5-8.5 simulations, respectively; "HG", "HA", "HT" denote the GHGs, AERs and NATs simulations, respectively. The numbers in brackets represent the number of ensemble members in the DAMIP simulations.
